# A bifactor representation of the Center for Epidemiological Studies Depression Scale for children: gender and age invariance and implications for adolescents’ social and academic adjustment

**DOI:** 10.1186/s13034-024-00717-z

**Published:** 2024-02-20

**Authors:** Yanhua Zhao, Jiahui Niu, Jing Huang, Yan Meng

**Affiliations:** 1 School of Psychology, Henan University, Jinming Campus, Kaifeng, 475003 P. R. China; 2 School of Education, Henan University, Jinming Campus, Kaifeng, 475003 P. R. China

**Keywords:** Bifactor, Center for epidemiological studies depression scale for children, Adolescence, Measurement invariance, Social and academic adjustment

## Abstract

**Background:**

The Center for Epidemiological Studies Depression Scale for Children (CES-DC) is a widely used scale for screening depressive symptoms in children and adolescents. This study aims to uncover the optimal factor structure of the DES-DC and presents an alternate conceptualization of adolescent depression by estimating bifactor models and several competing models using a sample of Chinese adolescents.

**Methods:**

The participants were 533 adolescents (49.7% boys, 49.7% girls, 3 participants did not report) between 12 and 18 years of age attending public secondary schools in the middle part of mainland China. Data were collected in classrooms using a questionnaire survey. A structural equation modeling approach was used to estimate and compare a series of competing models for the DES-DC.

**Results:**

A Bifactor exploratory structural equation model (Bi-ESEM) with the best model fit was retained for representing the current data. Tests of measurement invariance demonstrated strict measurement equivalence across gender and age. No gender and age differences have been found in the general depression factor. Findings provided evidence for the composite reliability and construct validity of DES-DC. Depressive symptoms positively related to the Big Five trait neuroticism, negative emotions, loneliness, social anxious behaviors, and disruptive behaviors during school and negatively related to agreeableness, conscientiousness, extraversion, physical health status, school connectedness, and academic self-efficacy.

**Conclusions:**

This study provides support for the one general factor construct of the CES-DC and the continuum concept structure of adolescent depression. Moreover, this research offers empirical evidence for comparing depression symptoms among adolescent populations with diverse genders and age groups. Additionally, the findings replicate and expand upon the implications of depressive symptoms on adolescents’ traits, well-being, social-relational adjustment, and academic adaptation.

**Supplementary Information:**

The online version contains supplementary material available at 10.1186/s13034-024-00717-z.

## Introduction

Depressive symptoms are common concerns in promoting adolescent mental health. The experience of depressive symptoms has negative effects on adolescents’ academic and social performances [1] and is relevant to adolescents’ behavioral problems [2] and suicide risks [3–5]. The prevalence rate of depressive symptoms has increased in the past years [6]. In a recent meta-analysis drawing from Chinese adolescent samples, the prevalence rate of depressive symptoms has been reported around 24.5-51.9% among secondary school students [7]. Despite the high prevalence rate of depressive symptoms, only a few of those suffering from depressive symptoms have been recognized or supported [8]. Effective prevention and intervention programs for adolescent depression are most likely to benefit from valid screening measures.

The Center for Epidemiologic Studies Depression Scale for Children (CES-DC) is a well-used and validated instrument in both primary care settings [9] and community settings [10, 11] and for adolescents with different cultural backgrounds [12]. The CES-DC was developed as the child version of the Center for Epidemiologic Studies Depression Scale [13], which has easier understanding expressions for children and adolescents aged 7–23 years old and is especially reliable for adolescents [14, 15]. Following the adult version [16], the CES-DC is composed of 20 items with a focus on depressive symptomology covering six important symptom clusters, including depressive mood, feelings of guilt and worthlessness, a sense of helplessness and hopelessness, psychomotor retardation, loss of appetite, and sleep disturbance. These selected symptoms are supposed to have four dimensions representing depressive affect (e.g., unhappy, lonely, sad), somatic symptoms (e.g., bothered, tired, poor sleep), interpersonal distress (unfriendly, dislike), and positive affect (e.g., good, hopeful, happy). The CES-DC has presented satisfactory internal consistency and test-retest reliability [12, 14]. In previous studies, these four dimensions have been occasionally treated as separate scales for assessing depressive symptoms, suggesting a multidimensional structure of depression [17]. Conversely, they have also been considered as one common construct [10], suggesting that depression is a unidimensional concept. These inconsistencies make the structure of CES-DC and the dimensionality of adolescent depression open question to answer.

### The internal structure of CES-DC

The original four-factor structure of CES-DC has been confirmed by previous studies using children and adolescent samples from Western [12] and non-Western societies [18, 19]. However, as shown in these studies, the factor intercorrelations were sizable, especially among the depressive affect, somatic symptoms, and interpersonal distress subscales, ranging from 0.64 to 0.93 (factor intercorrelations between the three negative subscales and positive affect ranges from 0.25 to 0.40). Additionally, three studies using exploratory factor analysis (EFA) provided different three-factor structures [20, 21] and a new four-factor structure for the CES-DC [22]. In these exploratory solutions, children and adolescents tend towards reporting depressive affect together with somatic symptoms; reporting depressive affect together with interpersonal distress; or reporting somatic symptoms together with interpersonal distress. In other studies involving adolescent samples from diverse cultural backgrounds, researchers observed similar trends of different symptom dimensions intertwining with each other [23–26]. Meanwhile, Olsson and Von Knoring (1997) [22] also reported high correlations between each item and the total score of the scale (M = 0.64, range = 0.48-0.80) in 16 items representing three negative symptoms subscales, and relatively lower correlations (M = 0.45, range = 0.38-0.60) in four positively stated items. As presented in the aforementioned studies, the high factor intercorrelations, significant item loadings on unintended factors, and strong item-to-total score correlations seem to hold theoretical significance, considering that depression is presumed to be a multidimensional construct composed of distinct facets that share some degree of conceptual overlap. Nonetheless, these conflicting results also underscore the necessity of reassessing the multidimensionality of the CES-DC and estimating a hierarchical conceptual structure for depression in children and adolescents.

Methodologically, while the total scale score was frequently employed to signify the degree of depressive symptoms, there was limited evidence supporting a unidimensional structure of the CES-DC. According to Reise, Bonfiay, and Haviland (2013) [27], the correlated trait model using CFA actually supports the use of subscale scores but not the total score. The presentation of a higher-order factor is a potential approach to assess the shared measurement trait. While earlier studies utilizing the adult version of CES-DC for adolescents have suggested a higher-order factor structure [28], the higher-order factor models for the CES-DC have not yet been investigated. The bifactor measurement model provides a possible way to test whether the items designed to represent specific dimensions can also be interpreted by a latent common construct [29], and helps to explain the question of “to what degree do total scale scores reflect reliable variation on a single construct” (p. 130, [27]). However, evidence for a common structure of the CES-DC obtained using bi-factor modeling is still lacking. Recently, Gomez and McLaren (2014) [30] provided a bifactor solution for the adult version of this scale, the Center for Epidemiological Studies Depression Scale (CES-D). They found that the majority of items in the CES-D loaded higher on the general factor (G-factor) than on the specific factors (S-factors) except the items for positive affect. Thus, to obtain the optimal structure for the CES-DC, the unidimensional, higher-order, and bifactor CFA (Bi-CFA) models were all examined and compared as competing models to represent the current data.

To examine these competing models, an exploratory structural equation modeling (ESEM) approach was applied. In the popularly used confirmatory factor analysis (CFA) models, items are only allowed to load on their target factors, with the cross-loadings on other factors constrained to be zero, which may potentially bias the estimation of factor correlations [31, 32]. In ESEM models, items are allowed to load on their target factor and cross-load on nontarget factors, which is more rational in analyzing the construct-relevant multidimensionality of the CES-DC. Compared with the CFA, ESEM generally produced lower factor correlations, better factor discriminant validity, and improved model fit [33]. Bi-ESEM (bifactor exploratory structural equation modeling) is a combined framework of the traditional bifactor model and the ESEM approach of factor analysis, which is a promising approach to investigating multidimensional measures originally designed to capture a hierarchically superior construct [31]. In the current study, a Bi-ESEM model was specified based on the framework proposed by Morin et al. (2016) [31], with all items loading on a general depression factor as well as on their target-specific domain factors. Several studies have supported the usefulness of Bi-ESEM in investigating the internal structure of multidimensional scale targeting at one hierarchical construct [34–36]. In addition, we estimated a correlated four-factor ESEM model and a higher-order ESEM model, together with the correlated CFA, the higher-order CFA, and the Bi-CFA models. We supposed that the Bi-ESEM model could outperform other competing models and show the best model fit.

### Measurement invariance across gender and age

It is very common to compare the depressive symptoms of teenagers of different genders and age groups. Before conducting these comparisons, it is crucial to initially establish the measurement invariance of the scale within these gender or age groups. This ensures that any disparities observed are not the result of measurement bias [37]. In previous research employing the adult version of CES-D to assess depressive symptoms in adolescents, evidence has supported various structures, including the four-factor structure [38, 39], different three-factor structures [25, 26, 40], and a higher-order factor structure [28], all with measurement invariance across gender. Regarding CES-DC, only Essau et al.‘s [18] study presented structure invariance of the a priori four-factor structure across gender. Thus, another objective of this study is to examine the measurement invariance across gender for the proposed Bi-ESEM structure. Specifically, we tested whether different gender groups responded to the scale in the same way and exhibited similar levels of latent means for the G-factor and S-factors. Concerning gender differences in depressive symptoms, a previous meta-analysis suggests that girls tend to report higher levels of depressive symptoms than boys [41]. In studies validating the CES-DC, girls generally report higher total scale scores and subscale scores for depressive affect and somatic symptoms compared to boys [18, 20, 42]. Yet, research on Chinese adolescents shows varied results; some studies find girls reporting more depressive symptoms [43–45], while a recent meta-analysis indicates no significant gender differences [7]. Further exploration of measurement invariance across gender and the latent mean differences of the factors in CES-DC will provide new insights into understanding depressive symptoms among boys and girls.

In terms of age consistency, researchers have found that when applying the CES-D to different age groups of adolescents, the structure of CES-D demonstrates equivalence across age groups [38]. Essau et al. [18] applied the CES-DC to assess the four-factor structure’s consistency across age stages and confirmed the age equivalence of CES-DC in early adolescents and late adolescents. Moreover, several studies have also verified the longitudinal cross-time measurement invariance of CES-D within adolescent populations [26, 46, 47]. Regarding the age difference, previous studies have found that the prevalence rate of depressive symptoms among late adolescents is higher than that of younger adolescents [7, 44, 48]. Using CES-DC, many studies provide evidence for the higher levels of depressive symptoms in late adolescence than in early adolescence across different cultures [18, 20, 49], but also studies reporting no age difference [42]. In studies using Chinese samples, higher-grade adolescents have a higher prevalence rate of depressive symptoms than the lower grade in secondary schools (see meta-analysis, [7]). In the present study, we also estimated the latent mean differences of the CES-DC across different age groups.

### Convergent and discriminant validity

To assess the convergent and discriminant validity of the CES-DC, we first investigated the associations of depressive symptoms with the Big Five personality traits. Previous studies find that the unique Big Five trait neuroticism is positively linked with depressive symptoms, whereas agreeableness, conscientiousness, and extraversion are negatively correlated with depressive symptoms, and openness is either negatively associated or not associated with depressive symptoms among Chinese adolescent samples [50, 51]. Therefore, we anticipated similar patterns in our study. Next, we examined the connections between depressive symptoms and various indicators of adolescents’ emotional and physical well-being, social-relational adjustment, and academic adaptations. Past studies have revealed that depressive symptoms tend to coincide with heightened negative emotions and reduced positive feelings [52, 53]. We predicted that individuals with more pronounced depressive experiences would report more negative and fewer positive emotions. Depressive symptoms have also been linked to poorer physical health concurrently and longitudinally [54, 55], so we expected a negative relationship between these symptoms and physical health status. Regarding social-relational adjustment, our expectations were based on prior research, suggesting that depressive symptoms are negatively associated with adolescents’ school connectedness and positively correlated with feelings of loneliness and social anxious behaviors [56–61]. We also explored the impact of depressive symptoms on adolescents’ patterns of adaptive learning. Some studies have indicated that higher depressive symptom scores are associated with lower general efficacy and academic efficacy, while others have linked depressive symptoms to disruptive behavior problems in school settings [62–65]. Thus, we hypothesized that adolescents with higher scores on depressive symptoms would report lower academic self-efficacy and higher disruptive behavior scores.

### The present study

The present study aimed to provide further evidence on the psychometric properties of the CES-DC using a Chinese adolescent sample and to support the usefulness of Bi-ESEM approach in investigating the multidimensionality of the scale targeting one common construct. Firstly, we attempt to examine the internal structure of the CES-DC by assessing whether a Bi-ESEM model fits the data better than other competing models. Moreover, we try to assess whether the factors in the retained Bi-ESEM model have satisfied reliability, factor loadings, explained common variances (EVC), and measurement invariance and latent mean differences across gender and age. Finally, we seek to provide evidence for the convergent and discriminant validity of the CES-DC in terms of adolescents’ personality, well-being, social-relational adjustment, and academic adaptations.

## Method

### Participants and procedure

Participants were 533 adolescents (49.7% boys, 49.7% girls, 3 participants did not report) between 12 and 18 years of age attending public secondary schools in the middle part of mainland China. Three responses that exhibited patterns and one response with 30% missing data were excluded. We then computed the Mahalanobis distance and its *p*-value for the CES-DC of the remaining 529 participants. As there were no participants with a *p*-value < 0.001, all 529 participants were retained. Among these 529 adolescents (M_*age*_ = 15.66, SD = 1.66), 49.9% were boys, 49.5% were girls, 3 participants did not report their gender. There were 27.9% of participants aged 12–14 years old, 29.3% aged 15–16, and 42.8% aged 17–18. Among the participants, 93.3% of them reported their ethnicity as Han Chinese, while 6.7% identified as a minority. Regarding the father’s education level, 32% had completed middle school or lower, 41.8% had completed high school or technical secondary school, and 26.2% had attended college or higher education institutions. This study received approval from the School Committees of two secondary schools and the Human Participants and Medical Ethics Committee of the authors’ university.

The short questionnaire survey, including the Chinese version of the CES-DC and demographic questions, was conducted after regular school hours and took approximately 10–15 min to complete. Two or three days later, 282 students (44% boys) who had participated in the short survey also participated in the longer questionnaire survey, which included all the scales mentioned in the following measurement section except the CES-DC. Every student present on the screening days was offered the opportunity to join the study. They were explicitly informed that their participation was entirely voluntary and that they could withdraw from the survey at any time. Following the completion of the informed consent process, participants proceeded to complete the questionnaires. As an acknowledgment of their participation, they received a stationery gift each time they submitted their questionnaires.

### Measurement

#### The Chinese version of the CES-DC

The 20-item English version of the CES-DC was originally translated into Chinese following a translation back-translation procedure. In the translation process, two bilingual psychologists independently translated the English version of the DES-DC into Chinese. They then collaborated to create a consensus version. This Chinese version was back-translated into English by a proficient translator who is also an English teacher. Discrepancies between the back-translated English version and the original were resolved through discussions with all translators. Afterward, we produced a preliminary test version, which we used in an initial test with 10 junior high school students. Their feedback on sentence revisions was incorporated to create the final version. According to the original validity study, this self-report scale was supposed to have four dimensions representing depressive affect, somatic symptoms, interpersonal distress, and positive affect. Items’ memberships in the original scale are presented in Table 1. This scale measured the occurrence frequency of each depressive symptom during the past week (e.g., “I felt down and unhappy this week”). Participants rated each item on a 4-point scale from 1 (*not at all*) to 4 (*a lot*). Four items of positive affect were negatively worded items.

**Table 1:** Standardized factor loadings (λ) and uniquenesses (δ) for the Bi-CFA and Bi-ESEM models

Indicator	Bi-CFA				Bi-ESEM					
	G λ	S λ	δ		G λ (ω_hs_)	S-DA λ	S-SS λ	S-ID λ	S-PA λ	δ
Depressive Affect (ω)		(0.90)			(0.03)	(0.90)				
Item 3 Blues	**0.62**	0.07	0.46		**0.63**	**0.07**	− 0.06	− 0.10	− 0.10	0.57
Item 6 Depressed	**0.79**	0.04	0.53		**0.76**	**0.13**	0.11	0.01	− 0.09	0.39
Item 9 Failure	**0.76**	− 0.22	0.22		**0.68**	**− 0.12**	0.03	0.30	− 0.21	0.39
Item 10 Fearful	**0.68**	− 0.11	− 0.38		**0.70**	**− 0.17**	− 0.12	0.19	0.05	0.43
Item 14 Lonely	**0.72**	0.15	0.45		**0.69**	**0.26**	0.01	0.14	− 0.02	0.44
Item 17 Crying	**0.58**	0.36	0.76		**0.59**	**0.38**	− 0.11	0.08	0.08	0.48
Item 18 Sad	**0.59**	0.66	0.84		**0.77**	**0.56**	− 0.01	0.02	− 0.04	0.09
Somatic symptoms (ω)		(0.76)			(0.02)		(0.76)			
item 1 Bothered	**0.49**	0.03	0.77		**0.49**	0.17	**− 0.01**	− 0.12	− 0.01	0.72
item 2 Appetite	**0.40**	0.01	0.86		**0.49**	− 0.11	**− 0.23**	− 0.41	0.08	0.52
item 5 Concentrate	**0.49**	0.55	0.38		**0.55**	− 0.07	**0.52**	− 0.13	0.05	0.40
item 7 Tired	**0.67**	0.29	0.61		**0.68**	− 0.01	**0.25**	0.01	− 0.06	0.47
item 11 Insomnia	**0.48**	− 0.01	0.37		**0.54**	− 0.12	**− 0.16**	− 0.09	0.08	0.65
item 13 Withdrawal	**0.37**	− 0.06	0.37		**0.36**	0.22	**− 0.03**	− 0.03	0.11	0.81
item 20 Get going	**0.54**	0.57	0.53		**0.59**	− 0.03	**0.47**	0.01	0.09	0.42
Interpersonal distress (ω)		(0.78)			(0.24)			(0.77)		
Item 15 Rejected	**0.62**	0.99	0.46		**0.62**	− 0.01	0.07	**0.32**	0.06	0.50
Item 19 Disliked	**0.72**	0.19	0.47		**0.69**	0.03	0.08	**0.46**	0.05	0.30
Positive affect (ω)		(0.83)			(0.59)				(0.82)	
item 4 Good	**− 0.29**	0.50	0.76		**− 0.28**	− 0.01	0.15	− 0.03	**0.51**	0.64
item 8 Hopeful	**− 0.32**	0.51	0.84		**− 0.32**	0.14	0.08	− 0.12	**0.52**	0.59
item 12 Happy	**− 0.41**	0.70	0.46		**− 0.40**	− 0.06	− 0.08	0.07	**0.72**	0.31
item 16 Enjoyed	**− 0.51**	0.72	0.47		**− 0.50**	− 0.03	− 0.06	0.15	**0.73**	0.19
ω_h_	(0.75)				(0.77)					

*Note.* Bi = bifactor; CFA = confirmatory factor analysis; ESEM = exploratory structural equation model; ω = McDonald’s omega coefficient; ω_h_ = McDonald’s hierarchical omega coefficient; G = general factor; S = specific factor; DA = depressive affect; SS = somatic symptoms; ID = interpersonal distress; PA = positive affect

#### The Chinese big five personality inventory brief version (CBF-PI-B)

The 15-item CBF-PI-B [66] evaluating the five personality dimensions was responded to on a scale from 1 *(disagree strongly)* to 6 *(agree strongly)*. The Cronbach’s alpha (α) coefficients in this sample were 0.66 for agreeableness, 0.60 for conscientiousness, 0.84 for openness, 0.67 for extraversion, and 0.75 for neuroticism. The average inter-item correlations (AIC) were assessed for agreeableness (AIC = 0.41), conscientiousness (AIC = 0.28), and extraversion (AIC = 0.40), which have shown α values lower than 0.70.

#### The positive and negative affect scale (PANAS)

Participants’ positive and negative emotional experiences were measured by the 20-item PANAS [67]. On a scale from 1 (*very slightly or not at all*) to 5 (*extremely*), respondents indicated their general positive emotions and negative emotions in the past four weeks (α = 0.86 for positive emotions; α = 0.85 for negative emotions).

#### The physical component of the shorter-form health survey (SF-12)

Participants’ general physical health status was measured using the physical component items from the SF-12 [68]. The six items asking participants’ general health, role physical, physical functioning, and bodily pain were responded to different scales. The item scores were transformed to a 0-100 scale. A composite score was generated by summing up the item scores, with a higher value representing better physical health status (α = 0.71).

#### The psychological sense of school membership scale (PSSM)

Participants were asked to report their connectedness with their schools using PSSM [69] on a scale from 1 (*not at all true*) to 5 (*completely true*). Item scores were summed, with a higher score indicating greater school connectedness (α = 0.88).

#### The three-item loneliness scale (UCLA-3)

Participants’ feeling of loneliness were measured by the UCLA-3 [70]. The three-item scale assessing self-reported feelings of lacking companionship, being left out, and being isolated was rated from 1 (*never*) to 4 (*often*) (α = 0.76).

#### The fear of social interaction scale (FSI)

Participants’ social anxious behaviors were measured using the FSI from the Chinese version of the Liebowitz Social Anxiety Scale [71]. The 11-item scale assessing adolescents’ feelings of fear in 11 different social interactional situations was rated on a scale from 1 (*not at all*) to 4 (*very much*) (α = 0.85).

#### The academic efficacy scale (AES)

Participants’ academic self-efficacy was assessed using the AES developed by Midgley and colleagues (2000) [72]. The five-item scale assessing participants’ perceptions of their competence in doing classwork was responded to on a scale ranging from 1 (*do not agree*) to 5 (*totally agree*) (α = 0.91).

#### The disruptive behavior scale (DBS)

Participants’ disturbing behaviors during class were measured using the DBS developed by Midgley and colleagues (2000) [72]. The five-item scale asking students to report their engagement in behaviors disturbing the classroom was answered on a scale ranging from 1 (*do not agree*) to 5 (*totally agree*) (α = 0.91).

### Data analyses

**Fig. 1:**
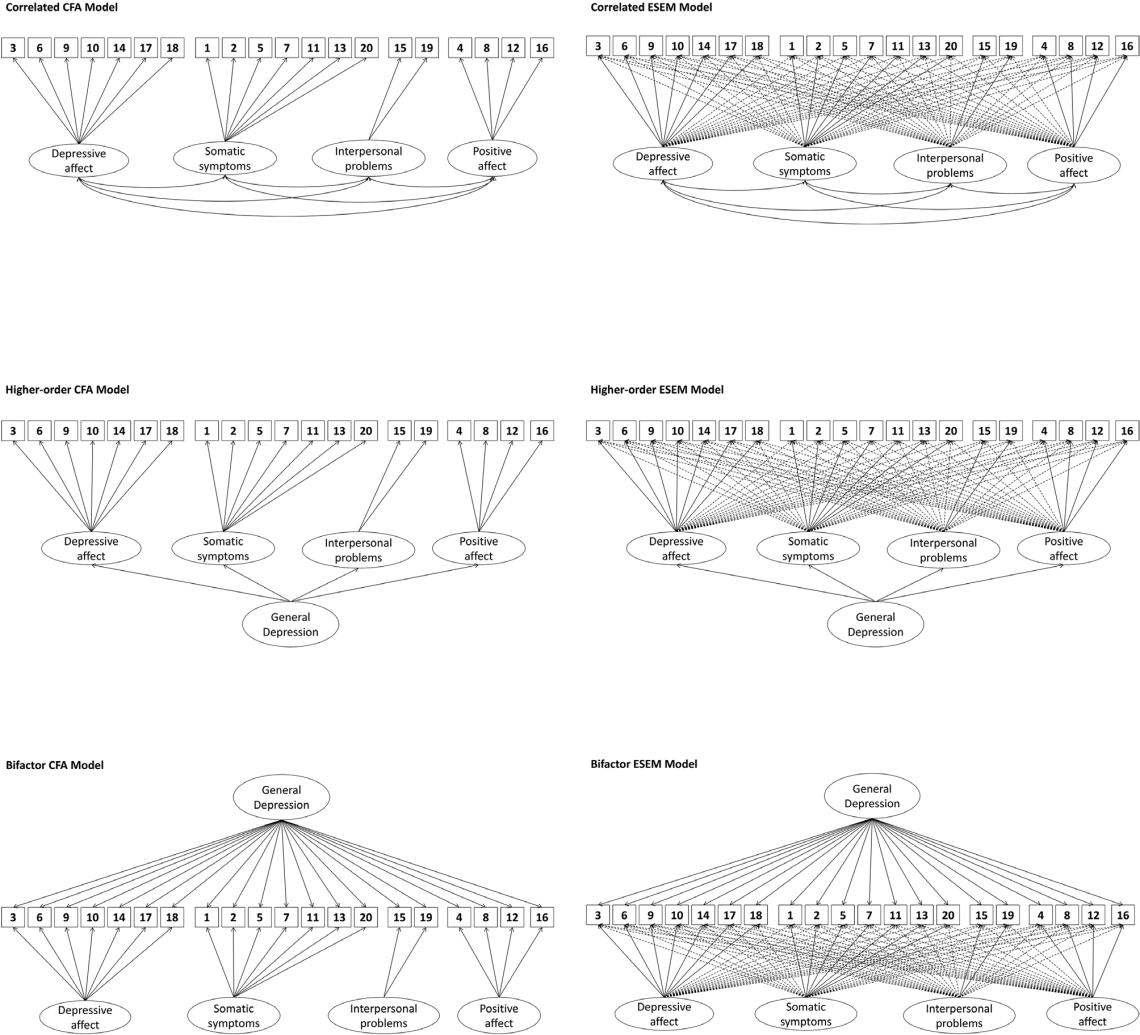
Graphical representation of the competing models examined in the study. Note. CFA = confirmatory factor analysis; ESEM = exploratory structural equation model; 1 = Bothered, 2 = Appetite, 3 = Blues, 4 = Good, 5 = Concentrate, 6 = Depressed, 7 = Tired, 8 = Hopeful, 9 = Failure, 10 = Fearful, 11 = Insomnia, 12 = Happy, 13 = Withdrawal, 14 = Lonely, 15 = Rejected, 16 = Enjoyed, 17 = Crying, 18 = Sad, 19 = Disliked, 20 = Get going

All statistical analyses were conducted with SPSS19 and *Mplus* 7.4. In the current sample, less than 1% percent of the values were missing in the CES-DC items. Given that our data were ordinal and multivariate nonnormal, a weighted least squares mean (WLSMV) estimator was used to estimate models and manage missing data [32]. The fit of the unidimensional model was estimated first, followed by the estimation of the correlated traits model, higher-order model, Bi-CFA model, and Bi-ESEM model. We conducted the model estimations in the following steps: (1) all items were specified to load on one depression factor, the model fit for the unidimensional structure was obtained; (2) to specify the correlated factor CFA model, each item was allowed to load on a priori factor which the item was originally designed to measure, and the correlations among four factors were freely estimated; (3) in the higher-order CFA model, a latent depression factor was added in the model and the four original factors were specified to present this higher-order depression factor; (4) in the Bi-CFA model, a general depression factor was added in the model, and each item was specified to load on both of the G-factor and its target factor; (5) to estimate the four-factor correlated factor ESEM model, higher-order factor ESEM model, and the Bi-ESEM model, items were specified to load on their target factors as done in CFA model, with allowing the rest items to load on nontarget factors as well (the factor loadings on nontarget factors were set to be close to zero). Figure 1 is a graphical representation of these competing models examined in this study.

Measurement invariances and latent mean differences across gender and age were tested for the proposed Bi-ESEM model. Three age groups (12–14, 15–16, 17–18) were built for testing measurement invariance. Three commonly used fit indices were selected to determine the fit of models: the comparative fit index (CFI), the Tucker-Lewis index (TLI), and the root mean square error of approximation (RMSEA). CFI and TLI values of 0.95 or greater reflect a good model fit to the data, whereas RMSEA values of 0.06 or less reflect a good fit to the data [73]. In model comparison, a change of CFI smaller than 0.01 and RMSEA smaller than 0.015 signify a non-significant change in the model fit [74].

McDonald’s (1999) [75] coefficient omega (ω) and coefficient omega hierarchical (ω_h_) were estimated to address the reliability of the common depression factor and the specific factors. After controlling for the general depression factor, the omega hierarchical for subscales (ω_hs_) was also calculated. According to Zinbarg et al. (2005) [76], a higher ω_h_ for the G-factor justifies a summation of the item scores in the scale, and a higher ω_hs_ for a specific factor justifies a summation of the item scores of the specific scale. To test whether a bifactor model represents the internal structure of DES-DC better than other alternative models, the explained common variance (ECV) was calculated to estimate the amount of variance explained by the G-factor [77]. Additionally, the percent of uncontaminated correlations (PUC) values were also calculated. A PUC > 0.80 or PUC < 0.80 but ECV > 0.60 and ω_h_ > 0.70 suggests the exhibition of some multidimensionality is not severe enough to distort the unidimensional structure of the scale [78]. To test the convergent and discriminant validity, variables representing adolescents’ personality, well-being, social-relational adjustment, and academic adaptations were correlated with observed depressive symptoms.

## Results

### Internal structure

The model fit statistics for the proposed models are reported in Table 2. As expected, the model fit for the unidimensional model was not adequate; the model fit for the correlated traits CFA model was adequate; the Bi-CFA model fitted the data better than the correlated factor and higher-order CFA models. Using an ESEM approach, the correlated factor, higher-order, and bifactor models fitted the data better than their corresponding CFA models. Regarding the correlations between factors, the correlated factor model in the ESEM approach revealed reduced inter-factor factor correlations compared with that in the CFA approach (See Table S1 of the supplementary materials 1). Overall, the Bi-CFA and Bi-ESEM models exhibited excellent fit, as evidenced by the CFIs and TLIs exceeding 0.95 and RMSEAs smaller than 0.06 [73]. Compared with the Bi-CFA model, the Bi-ESEM model showed an improved CFI value of 0.13 and a decreased RMSEA value of 0.002, which exceeded the cutoff value of 0.01 for CFI change [74], indicating a better fit. Therefore, the Bi-ESEM was retained for further analyses as the best structural model of the data.

**Table 2:** Model fit statistics for factor analyses and measurement invariance testing

Models	χ2	df	CFI	TLI	RMSEA	[90% CI]	ΔCFI	ΔRMSEA
Unidimensional Model	1374.495	170	0.834	0.814	0.118	[0.112-0.123]		
CFA								
Correlated traits	545.109	164	0.947	0.939	0.067	[0.061-0.074]	0.113	− 0.051
Higher-order CFA	538.294	166	0.949	0.941	0.066	[0.060-0.072]	0.002	− 0.001
Bi-CFA	399.587	151	0.966	0.957	0.057	[0.050-0.063]	0.016	− 0.009
ESEM								
Correlated traits	332.135	116	0.970	0.951	0.060	[0.053-0.068]		
Higher-order ESEM	328.117	118	0.971	0.953	0.059	[0.051-0.067]	0.002	− 0.001
Bi-ESEM	254.136	100	0.979	0.960	0.055	[0.047-0.063]	0.007	− 0.004
Models	χ2	*df*	CFI	TLI	RMSEA	[90% CI]	ΔCFI	ΔRMSEA
Invariance across gender								
Configural	344.536	215	0.980	0.965	0.049	[0.039-0.058]		
Scalar invariance	447.846	310	0.979	0.975	0.042	[0.033-0.050]	− 0.001	− 0.007
Strict invariance	462.385	330	0.980	0.977	0.040	[0.031-0.048]	0.001	− 0.002
Latent means invariance	584.676	335	0.962	0.957	0.054	[0.047-0.061]	0.018	0.014
Invariance across age (12–14, 15–16, 17–18)								
Configural	462.981	330	0.981	0.967	0.049	[0.038-0.059]		
Scalar invariance	661.315	520	0.979	0.978	0.040	[0.030-0.049]	0.002	− 0.009
Strict invariance	718.436	560	0.977	0.977	0.041	[0.031-0.049]	− 0.002	0.001
Latent means invariance	787.315	570	0.968	0.968	0.047	[0.039-0.055]	0.009	0.006

*Note.* χ^2^ = chi square (weighted least square estimator was used); *df* = degrees of freedom; CFI = Comparative fit index; TLI = Tucker-Levis Index; RMSEA = root mean square error of approximation; 90% CI = 90% confidence interval for the RMSEA; CFA = confirmatory factor analysis; ESEM = exploratory structural equation model; Bi = bifactor

Factor loadings of the G- and S-factors for Bi-ESEM and Bi-CFA models were reported in Table 1. In the Bi-ESEM model, the mean target loadings for the G-factor were adequate (|λ_mean_|= 0.57). For S-factors, factor positive affect exhibited mean target loadings at 0.62 and minimum loadings greater than 0.50, factor interpersonal distress exhibited mean target loadings at 0.39 and minimum loadings greater than 0.30, the mean target loadings for factor depressive affect and somatic symptoms were around 0.24, and only two depressive affect items and two somatic symptoms items out of seven items exhibited salient loadings higher than 0.30. Comparing with the item loadings for specific factor depressive affect, somatic symptoms, and interpersonal distress, all the item loadings for G-factor were bigger, and all these loadings were greater than 0.30. For factor positive affect, although four target item loadings for G-factor were smaller than for specific positive affect factor, three of four items loadings for G-factor exceeded 0.30, (except item 4, |λ| = 0.28). Therefore, almost all items were salient measures of the G-factor, suggesting a well-defined general construct. The ω value for the G-factor (ω_h_ = 0.77) and S-factors (ω_s_ > 0.76) were adequate. The ω values for S-factors (ω_hs_) were marginal when the G-factor was controlled, with 0.03 for depressive affect, 0.02 for somatic symptoms, and 0.24 for interpersonal distress, except positive affect, with a moderate value of 0.59. The ω_h_ value for the G-factor was above the preferred value 0.75 [78], the ω_hs_ values are smaller than that for the G-factor, justifying a reliable G-factor and the summation of the items scores of the CES-DC [76].

The Bi-ESEM model explained 88.4% of the total score variance. The G-factor accounted for 67.1% of the common variance, and the S-factors accounted for the remaining 32.9%. Among S-factors, depressive affect, somatic symptoms, interpersonal distress, and positive affect were explained for 5.87%, 7.0%, 3.8%, and 16.2%, respectively, of the common variance. The PUC value was 0.74 (< 0.80), the ECV value was 0.67 (> 0.60) and ω_h_ was 0.77 (> 0.70) for the general factor, suggesting that the exhibition of some multidimensionality is not severe enough to inflate the unidimensional structure of the scale [27].

### Measurement invariance and latent mean differences across gender and age

The measurement invariance tests indicated that the configural, scalar (factor loadings and thresholds invariance), and strict models (factor loadings, thresholds, and.

errors invariance) for gender and age groups showed adequate fit to the data (see Table 2). In comparison with configural models for gender and age groups, no significant change of CFIs was detected in scalar models or strict models. Compared with the configural model for gender groups, the strict model showed a decreased RMSEA value at 0.009 and no decrease fit of CFI value. Compared with the configural model for age groups, the strict model showed a decreased CFI value of 0.004 and a decreased RMSEA value of 0.008. These changes in CFI and RMSEA were respectively lower than the cutoff of 0.01 and 0.015 [74], providing evidence for an invariant structure of the CES-DC across gender and age groups.

Based on the invariant structure of the Bi-ESEM model, the latent mean differences were estimated (see Table 3). The latent mean invariance model presents a good fit for both gender and age groups (see Table 2). As for gender, results showed a decreased fit of the latent mean invariance model in comparison with the strict model (ΔCFI = 0.018, ΔRMSEA = 0.014), the change of the CFI value was bigger than the cutoff 0.01, and the change of the RMSEA was close to the cutoff 0.015 [74], suggesting significant differences among latent factor means. Specifically, no gender difference has been found in the general depression factor. Compared with boys (means of males were set to be zero), girls presented lower latent means in somatic symptoms, interpersonal distress, and positive affect, but higher means in the depressive affect. As for age, results showed that the change of the CFI and RMSEA values (ΔRMSEA = 0.005, ΔCFI = 0.006) were respectively lower than the cutoff values of 0.01 and 0.015 when the latent mean invariance model was compared with the scalar model, suggesting a nonequivalent trend of the latent means. Age group analyses revealed that middle (15–16) and late adolescents (17–18) reported higher latent means of somatic symptoms than early adolescents (12–14). Late adolescents reported higher interpersonal distress than middle and early adolescents but reported a lower latent mean of positive affect. No significant age difference has been found in the general depression factor.

**Table 3:** Latent mean tests across gender and age groups

Factor	Gender Groups		Age Groups										
	Mean male	Mean female		Mean 12–14	Mean 15–16	Mean 17–18	Mean 12–14	Mean 15–16	Mean 17–18	Mean 12–14	Mean 15–16	Mean 17–18	
General factor	0.00	− 0.16 (0.13)		0.00	− 0.07 (0.66)	0.12 (0.38)	0.05 (0.67)	0.00	0.18 (0.12)	− 0.11 (0.37)	− 0.20 (0.12)	0.00	
Depressive affect	0.00	0.47* (0.00)		0.00	0.16 (0.51)	0.30 (0.30)	− 0.12 (0.52)	0.00	0.15 (0.52)	− 0.24 (0.33)	− 0.15 (0.52)	0.00	
Somatic symptoms	0.00	− 0.41** (0.00)		0.00	0.52** (0.00)	0.66** (0.00)	− 0.59** (0.00)	0.00	− 0.03 (0.88)	− 0.56** (0.00)	0.02 (0.87)	0.00	
Interpersonal distress	0.00	− 0.33** (0.02)		0.00	− 0.09 (0.71)	0.57* (0.03)	0.09 (0.72)	0.00	0.67** (0.00)	− 0.56* (0.02)	− 0.62** (0.00)	0.00	
Positive affect	0.00	− 0.67** (0.00)		0.00	− 0.25 (0.20)	− 0.46* (0.01)	0.16 (0.18)	0.00	− 0.22 (0.13)	0.31** (0.01)	0.22 (0.12)	0.00	

*Note.* * *p* < .05, ** *p* < .01

### Convergent and discriminant validity

Table 4 presents the correlations between observed depressive symptoms and the variables used for testing convergent and discriminant validity. Depressive symptoms were strongly and positively correlated with neuroticism, negative emotional experience, and loneliness, and positively correlated with social anxious behaviors and disruptive behaviors during school, with medium to low effect sizes. The traits agreeableness, extraversion, and conscientiousness were negatively related to depressive symptoms, with low to medium effect sizes. No significant correlation has been observed between openness and depressive symptoms in this case. Higher levels of physical health status, academic self-efficacy, and school connectedness are moderately associated with fewer depressive symptoms.

**Table 4:** Correlations between depressive symptoms and variables used for testing convergent and discriminant validity (*N* = 282)

	Personality						Well-being			Social Relationships			Academic Adaptations	
	CES-DC (Total score)	Agreea-bleness	Conscien-tiousness	Open-ness	Extra-version	Neuro-ticism	Positive emotions	Negative emotions	Physical health status ^a^	School connected-ness	Loneliness	Social anxious behaviors	Academic self-efficacy	Disruptive behaviors
CES-DC (Total score)	1	− 0.13*	− 0.24***	− 0.10	− 0.17**	0.60***	− 0.28***	0.64***	− 0.36***	− 0.45***	0.56***	0.35***	− 0.37***	0.20***
*M*	1.85	3.84	3.55	3.40	3.37	2.83	3.12	2.08	73.82	3.71	1.73	2.02	3.53	1.64
*SD*	0.46	0.77	0.71	0.94	0.94	0.97	0.74	0.71	22.04	0.61	0.51	0.71	0.94	0.81

*Note.* ^*a*^ N = 278; * p < .05, ** p < .01, ***p < .001.

## Discussion

In the present study, we aimed to examine the factor structure of the CES-DC using a sample of Chinese adolescents and to validate a Chinese version of the CES-DC tailored for adolescents. We adopted an emerging analysis approach known as the Bi-ESEM model to investigate the concept structure of depression as measured by CES-DC. The original Bi-ESEM model with one G- and four S-factors provided the best model fit in comparison with the competing CFA and ESEM models. In simpler terms, different symptoms described as depressive affect, somatic symptoms, interpersonal distress, and positive affect represent one underlying depression dimension, supporting the continuum concept structure of depression.

### Internal structure

The priori four-factor structure confirmed by previous studies [18, 19] was also supported by this adolescent sample. The one-dimensional CFA model did not show an adequate fit to the data. The higher-order CFA and ESEM models showed a better model fit than the correlated CFA and ESEM model, and the Bi-CFA and Bi-ESEM models outperformed the corresponding higher-order models, supporting that bi-factor modeling is a useful approach in demonstrating a scale whose items explained by specific factors can also be explained by a common latent factor [29]. Consistent with Morin et al. (2016) [31], the Bi-ESEM factor model outperformed the Bi-CFA model and presented the best model fit to the data. As expected, majority factor loadings on the G-factor were higher than on S-factors except for the items for the positive affect subscale, which consisted of former studies reporting higher correlations between depressive affect, somatic symptoms, and interpersonal distress subscales but relatively lower correlations between positive affect and other three negative symptom subscales [12, 18]. Reliability analyses have revealed adequate composite reliability, ECV, and PUC values for the G-factor, while hierarchical reliability values for the S-factors are less meaningful. In the case of positive affect, although the variability of its items was more strongly explained by its intended factor rather than the general depression factor, the positive affect subscale (ω_h_ = 0.59) demonstrated a composite reliability below the desirable threshold of 0.70 after controlling for the general depression factor. Furthermore, there were no isolated indicators of positive affect with weak associations with the general factor. Hence, we are inclined to view raw scores on the CES-DC primarily as indicators of the general depression factor, with minimal influence from multidimensionality. Overall, the CES-DC predominantly represents a unidimensional concept of adolescent depression. This suggests that we can conceptualize the level of depression in adolescents as a continuous dimension, spanning from low to high intensity.

The Bi-ESEM model demonstrates an invariant measurement structure for CES-DC across gender and age groups. These results extend the prior findings that have indicated an invariant measurement structure for CES-DC [18] and CES-D in adolescent samples [18, 26, 28, 38, 39]. This suggests that the item scores and latent means obtained from different gender and age groups can be considered comparable in terms of meaning and response patterns. This allows for meaningful group comparisons related to depression using CES-DC. Consistent with previous research [7, 43, 44], the current sample demonstrates no significant gender difference in depression between boys and girls. Regarding positive affect, in line with earlier literature [26, 40], girls reported lower scores than boys. Contrary to existing studies [7, 48] that reported higher depressive symptom scores in late adolescents, no age difference was observed in the general depression factor. This finding aligns with the results of Tsocheva et al.‘s (2018) study [42]. Furthermore, in contrast to previous findings [18], late adolescents reported significantly lower levels of positive affect compared to early adolescents. These findings suggest that while certain symptoms may be a better marker of depression for specific groups [39], the latent levels of depression among boys and girls, as well as among early, middle, and late adolescents, are similar. This underscores the importance of giving equal attention to both genders and various age groups in research and practices aimed at preventing depression.

### Convergent and discriminant validity

This study builds upon and reinforces prior research regarding the implications of depressive symptoms across various functional domains. Consistent with earlier findings [51], we observed significant correlations between depressive symptoms and Big Five personality traits in the anticipated directions. Furthermore, our research affirmed that depressive symptoms predict a heightened experience of negative emotions, a reduced experience of positive emotions, and poorer physical health, aligning with previous studies [52–55]. These findings indicate that the CES-DC effectively captures individuals’ experiences of well-being and exhibits strong construct validity when linked to personality traits, emotional experiences, and physical symptoms. In the social functioning domain, our study replicated previous research by demonstrating a negative association between depressive symptoms and school connectedness and positive correlations between depressive symptoms and feelings of loneliness and social anxiety [56–61]. These findings suggest that adolescents with more depressive symptoms may encounter greater social and relational challenges and experience increased feelings of loneliness at school. Moreover, our findings aligned with earlier studies in indicating that individuals reporting higher depressive symptoms tended to report lower levels of academic self-efficacy [63] and displayed more disruptive behavior problems at school [64]. In summary, these results suggest that the emergence of depressive symptoms may predict adolescents’ characteristics, well-being, social-relational adjustment, and academic adaptation.

### Limitations and future research

There are several limitations to interpreting the findings of this study. First, the findings reported here are based on a population sample, which may not be considered representative of the sample differed in mental health states. Future studies validating the bifactor structure of the CES-DC using clinical samples are suggested. Second, the suggestion of taking the original DES-DC as a unidimensional structure was based on the statistical indices of the G-factor suggested by Reise et al. (2012) [78]. However, the utilization of subscale scores may hold practical significance for intervention and treatment. Specifically, although the composite reliability of positive affect was lower than the preferred value of 0.75 for deciding a reliable specific factor, it still accounted for a portion of the common variance. For practical applications, incorporating scores from the positive affect subscale alongside the total score may provide deeper insights into the understanding and interpretation of depression and its associated outcomes. Thirdly, the present study is constrained by the absence of a replication sample. By incorporating a replication sample, we can dismiss the likelihood that the better fit of the bi-ESEM model is merely by chance. Future replication studies would make the bi-factor structure representation of the CES-DC more convincing.

### Conclusion

In conclusion, the present findings support the unidimensional structure of CES-DC and the continuum concept structure of adolescent depression. It is recommended to continue using the total CES-DC score in future research. Moreover, the CES-DC exhibits measurement invariance across gender and age, establishing a basis for comparisons between different gender and age groups. Finally, the Chinese version of CES-DC proves to be a reliable and culturally adapted tool for screening depressive symptoms in Chinese-speaking adolescents, encouraging further cross-cultural comparisons and collaborative efforts in adolescent depression prevention.

### Electronic supplementary material

Below is the link to the electronic supplementary material.

Supplementary Material 1

## Data Availability

The data that support the findings of this study are available in this published article and its supplementary information files.
